# Independent position correction on tumor and lymph nodes; consequences for bladder cancer irradiation with two combined IMRT plans

**DOI:** 10.1186/1748-717X-5-53

**Published:** 2010-06-15

**Authors:** Dominique C van Rooijen, René Pool, Jeroen B van de Kamer, Maarten CCM Hulshof, Caro CE Koning, Arjan Bel

**Affiliations:** 1 Department of Radiation Oncology, Academic Medical Center, Amsterdam, The Netherlands

## Abstract

**Background:**

The application of lipiodol injections as markers around bladder tumors combined with the use of CBCT for image guidance enables daily on-line position correction based on the position of the bladder tumor. However, this might introduce the risk of underdosing the pelvic lymph nodes. In this study several correction strategies were compared.

**Methods:**

For this study set-up errors and tumor displacements for ten complete treatments were generated; both were based on the data of 10 bladder cancer patients. Besides, two IMRT plans were made for 20 patients, one for the elective field and a boost plan for the tumor. For each patient 10 complete treatments were simulated. For each treatment the dose was calculated without position correction (option 1), correction on bony anatomy (option 2), on tumor only (option 3) and separately on bone for the elective field (option 4). For each method we analyzed the D_99% _for the tumor, bladder and lymph nodes and the V_95% _for the small intestines, rectum, healthy part of the bladder and femoral heads.

**Results:**

CTV coverage was significantly lower with options 1 and 2. With option 3 the tumor coverage was not significantly different from the treatment plan. The ΔD_99% _(D_99%, option n _- D_99%, treatment plan_) for option 4 was small, but significant. For the lymph nodes the results from option 1 differed not significantly from the treatment plan. The median ΔD_99% _of the other options were small, but significant. ΔD_99% _for PTV_bladder _was small for options 1, 2 and 4, but decreased up to -8.5 Gy when option 3 was applied. Option 4 is the only method where the difference with the treatment plan never exceeds 2 Gy. The V_95% _for the rectum, femoral heads and small intestines was small in the treatment plan and this remained so after applying the correction options, indicating that no additional hot spots occurred.

**Conclusions:**

Applying independent position correction on bone for the elective field and on tumor for the boost separately gives on average the best target coverage, without introducing additional hot spots in the healthy tissue.

## Background

External beam radiotherapy is the treatment of choice for bladder cancer patients unfit for a radical cystectomy or willing to preserve their bladder function. Conventional radiotherapy generally consists of irradiation of the entire bladder. However, when the tumor is unifocal, a focal tumor boost has been shown to provide a high local control rate with acceptable toxicity [1,2]. In focal bladder cancer irradiation, however, the large day-to-day variation of the tumor position causes a major problem [3-8]. The implementation of image-guided radiotherapy (IGRT) and daily on-line position correction for unifocal bladder tumors will reduce the positional uncertainty and could enable margin reduction.

At our department, bladder tumor irradiation involves additional pelvic lymph node irradiation by an elective field. The movement of the lymph nodes with respect to the bony anatomy is relatively small [9] and is independent of the movement of the bladder. Therefore the implementation of on-line position correction for the bladder tumor might introduce the risk of underdosing the pelvic lymph nodes. A couple of studies have addressed this problem for the prostate and two possible correction methods are proposed.

Ludlum et al. have developed an algorithm that adjusts the position of the MLC leaves conformal to the prostate, while keeping the other leaves unchanged [10]. The rationale behind this correction method is that the table position correction does not have to be applied for the tumor and bone separately. Unfortunately, it is currently not possible to adjust the leaves during treatment.

Rossi et al. show that a considerable degradation of the delivered dose to the pelvic lymph nodes might occur when on-line position correction is applied based on the prostate position [11]. They propose to start the treatment with the execution of the boost plan. After a number of fractions, the uncertainty of the prostate position can be estimated and with that the PTV margin for the lymph nodes can be determined. For the bladder treatment used at our department this method is not an option, because the lymph nodes are being irradiated in almost all fractions. Hence, the uncertainty of the tumor position cannot be estimated before the treatment of the lymph nodes starts.

Our proposal is to make two treatment plans and correct them separately, despite the overhead of additional image analysis and possible couch correction. The purpose of this study is to investigate if the plans can be separated and moved without losing either tumor or bladder and lymph node coverage. This correction strategy is compared with correction on bony anatomy, correction on tumor position and no position correction.

## Methods

### Patients and prescribed dose

This simulation study included 20 patients with a histologically proven bladder tumor who received a treatment at our department. Our current department policy is to prescribe 55 Gy if the tumor is close to the small intestines and 60 Gy if the small intestines are not at risk. Ten patients were given a prescribed dose of 55 Gy on the tumor and ten patients were given a prescribed dose of 60 Gy. For all patients an elective dose of 40 Gy was prescribed to the lymph nodes and healthy part of the bladder. The patients were treated with a full bladder. They were instructed to void the bladder and drink 250 cc of water one hour before the treatment.

All patients were actually treated with our current technique [1]. The patients who were treated with 55 Gy, received 20 fractions of 2 Gy to the elective field and a concomitant boost of 0.75 Gy to the tumor. The patients who were treated with 60 Gy, received the same schedule as the 55 Gy patients in the first 20 fractions, with two subsequent fractions of 2.5 Gy to the tumor.

### Delineation and treatment planning

For all patients a planning CT with 3 mm slices was acquired with the patient in supine position. Before the planning CT was acquired lipiodol was injected under cystoscopic guidance on 3 to 5 locations, thereby indicating the border of the tumor [12]. Lipiodol is a contrast medium that is visible on CT as well as on CBCT. The lipiodol guided the GTV delineation and it enabled on-line position verification. More details regarding the clinical application of the lipiodol injections were given by Pos et al. [12]. The lipiodol spots remained visible throughout the entire course of radiotherapy. The tumor was delineated by an experienced radiation oncologist. The delineated tumor volume was defined as CTV [13]. The bladder, rectum, pelvic lymph nodes, femoral heads and small bowel were delineated as well.

In consideration of daily on-line position correction, a CTV - PTV_tumor _margin of 5 mm and a lymph node (ln) - PTV_ln _margin of 5 mm were chosen [14]. Because the bladder volume has a substantial day-to-day variation we opted for a bladder - PTV_bladder _margin of 20 mm in the cranial and anterior direction and 10 mm in the posterior, lateral and caudal direction.

**Figure 1:**
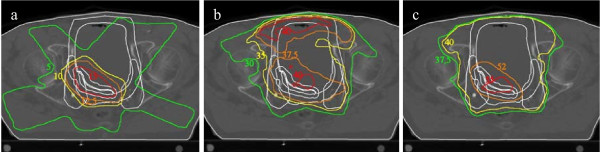
**Dose distributions. **An example of the dose distribution in Gy of a boost plan (a), an elective plan (b) and the composite plan (c) for one patient.

Intensity modulated radiotherapy (IMRT) plans were made with the planning system PLATO (Nucletron BV, Veenendaal, The Netherlands), using an energy of 10 MV. The following beam angles were used for each plan: 40°, 110°, 180°, 250° and 320°. Two separate IMRT plans were made. The first plan was the boost of 15 Gy to the tumor in 20 fractions and the second plan was 40 Gy to the elective field in 20 fractions. Both plans were administered in each fraction, with the option to adjust the patient position in between the execution of both plans. After 20 fractions, the patients with a prescribed dose of 60 Gy received an additional boost of 5 Gy on the tumor in 2 fractions. To prevent overdosage and hotspots, the dose of the boost plans was taken into account while making the elective plan. Figure 1 shows an example of the dose distribution of a boost plan, an elective plan and the composite dose distribution.

The requirement of the plans was that 99% of the volume of the target received 95% of the prescribed dose, which is 52.25 Gy or 57 Gy for the PTV_tumor _and 38 Gy for the PTV_bladder _and PTV_ln_.

### Simulation of tumor displacement and correction

**Figure 2:**
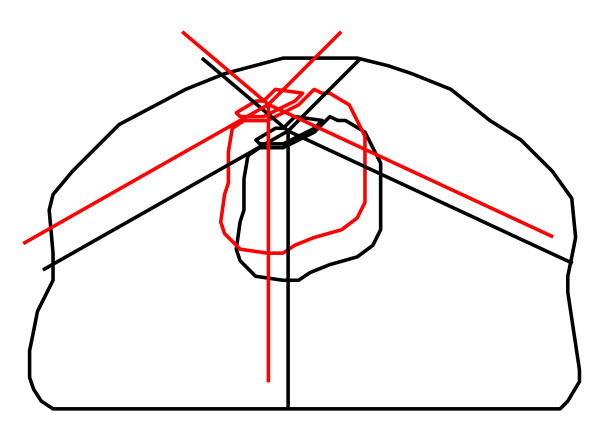
**Schematic representation of simulation. **The black lines represent a CT slice of the patient in the treatment planning situation. The red tumor represents the tumor after internal displacement. For analyzing the hot spots in the bladder, the bladder moves with the tumor. The red lines represent the treatment beams when position correction based on tumor position (option 3) is applied.

The lipiodol that was injected to guide the delineation of the tumor can also be used as marker for on-line position verification [15]. The set-up error and tumor displacement of ten bladder cancer patients with 5 to 9 CBCT scans were determined using XVI release 3.5 (Elekta, Crawley, UK) for the registration. The set-up error was the result of the match on the bony anatomy and the tumor displacement was the displacement of the tumor with respect to the bony anatomy. For each of the ten patients the mean set-up error (± sd) and the mean tumor displacement (± sd) were determined in each direction. From this, set-up errors and tumor displacements of ten complete treatments were generated using a Monte Carlo generator, assuming a Gaussian distribution. The generated distributions of deviations were applied for all 20 patients for whom IMRT plans were made, resulting in 200 simulated treatments. For the dose calculation the body was displaced with respect to the beams to simulate set-up errors. In addition, the delineated tumor was moved with respect to the bony anatomy to simulate tumor movement (figure 2). A full dose calculation was done for every fraction and afterwards the dose was summated for each organ separately. All reported results are therefore the results of a complete treatment. For each treatment, the dose distribution was calculated for the following four situations:

1. No position correction

2. Daily position correction based on the bone match for both plans

3. Daily position correction based on the tumor match for both plans

4. Daily position correction based on the bone match for the elective plan and based on the tumor match for the boost plan

Figure 2 shows an example of a simulated fraction. The position of the tumor has changed and position correction has been applied based on the tumor match (option 3). The dose distribution in this new situation was calculated. This was done for every treatment fraction.

A stand-alone version of PLATO's dose engine was used for the dose calculations [16]. This PC version of PLATO was highly optimized for fast dose calculations on a graphical card [17].

### Data analysis

For the bladder, it was less obvious to determine how the dose was affected by the four correction options. The bladder volume changes substantially, but these volume changes were not simulated. Figure 2 shows schematically what was simulated. To determine the hot spots in the bladder, the bladder was shifted with the tumor in the simulation. The rationale behind this was that the hot spots were expected to be near the tumor. In the case that the bladder was considered as a target, we analyzed the PTV_bladder_, because the PTV is supposed to cover the whole bladder and possible volume changes were incorporated in the margin.

## Results

### Tumor displacement data

For ten patients, the mean set-up error (± sd) and the mean tumor displacement (± sd) were determined for each main direction. The tumor displacement was determined with respect to the bony anatomy. The results for all patients are shown in table 1. Most of the systematic set-up errors were within 2 mm, with one exception of 4.4 mm. The results of the tumor registration showed more variation. The systematic tumor displacement ranged from 0.3 mm to 7.4 mm in a single direction. All simulations in this study were based on these displacement data.

**Table 1:** The match results of ten bladder cancer patients

Tumor	M_LR _(mm ± sd)	M_CC _(mm ± sd)	M_DV _(mm ± sd)	Vector length V (mm)
Patient 1	1.4 (± 1.1)	-1.4 (± 1.1)	-4.4 (± 1.6)	4.8

Patient 2	0.4 (± 0.7)	1.3 (± 2.6)	-7.4 (± 1.3)	7.5

Patient 3	-0.9 (± 1.4)	2.3 (± 1.9)	4.2 (± 4.5)	4.9

Patient 4	2.7 (± 1.0)	-5.9 (± 4.1)	0.5 (± 3.8)	6.5

Patient 5	0.3 (± 0.8)	-1.9 (± 1.8)	0.7 (± 1.8)	2.1

Patient 6	-0.4 (± 1.1)	-4.7 (± 3.3)	-1.5 (± 1.8)	4.9

Patient 7	2.4 (± 1.6)	5.0 (± 2.1)	3.5 (± 3.0)	6.6

Patient 8	2.4 (± 1.6)	-2.8 (± 4.6)	1.0 (± 3.3)	3.8

Patient 9	0.4 (± 2.5)	-6.0 (± 4.6)	-3.6 (± 2.4)	7.0

Patient 10	1.0 (± 0.9)	-1.6 (± 3.1)	6.5 (± 4.5)	6.8

Set-up				

Patient 1	0.6 (± 1.2)	1.0 (± 2.3)	1.6 (± 1.4)	

Patient 2	-0.9 (± 4.6)	-1.0 (± 2.2)	0.2 (± 3.4)	

Patient 3	-1.9 (± 2.0)	0.0 (± 0.9)	-2.5 (± 3.7)	

Patient 4	-0.8 (± 6.0)	0.6 (± 2.1)	-0.2 (± 2.3)	

Patient 5	2.1 (± 2.8)	-1.1 (± 1.8)	0.0 (± 2.1)	

Patient 6	-1.7 (± 3.9)	1.8 (± 1.1)	-1.3 (± 2.9)	

Patient 7	-2.7 (± 2.4)	-0.4 (± 1.0)	2.3 (± 1.4)	

Patient 8	-1.4 (± 3.5)	1.2 (± 4.6)	-2.3 (± 2.1)	

Patient 9	-1.4 (± 0.9)	-0.3 (± 1.3)	-4.4 (± 0.7)	

Patient 10	-2.1 (± 2.7)	0.8 (± 0.7)	-1.1 (± 0.7)	

The upper half of the table shows the results of the tumor registration. The lower half shows the results of the registration on bony anatomy. M_LR _is the mean in the left-right direction; M_CC _is the mean in the craniocaudal direction and M_DV _is the mean in the dorsoventral direction. The vector length V is the absolute tumor displacement and is defined as:

### Targets

**Figure 3:**
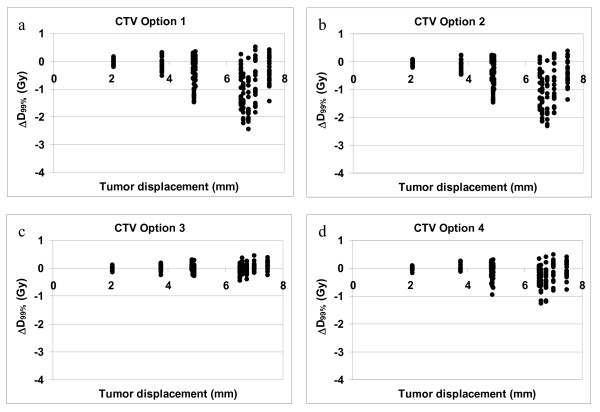
**CTV coverage. **These figures show the ΔD_99% _of the CTV versus the tumor displacement vector for the four correction strategies. Note that some of the tumor displacement vector lengths overlap (see table 1)

Because ΔD_99% _was not normally distributed we report the median ΔD_99% _(range) and the data were tested with the Wilcoxon signed rank test. For the CTV the correction based on tumor match (option 3) was the only strategy in which the D_99% _of the tumor was not statistically significant lower than in the treatment plan (p = 0.33). The median ΔD_99% _of this option was 0.01 Gy (range: -0.44 to 0.46). The D_99% _of all other treatment options was significantly lower (p < 0.001) than in the treatment plan (table 2). However, figure 3 shows that for option 4 most simulations resulted in a ΔD_99% _of less than 1.0 Gy, where for option 1 and 2 the ΔD_99% _exceeds 2.0 Gy in a number of simulations.

**Table 2:** The ΔD_99% _(D_99%, option n _- D_99%, treatment pla__n_) of the targets with the four correction options

	Option 1	Option 2	Option 3	Option 4
GTV	-0.41 Gy * (-2.44 - 0.51)	-0.45 Gy * (-2.32 - 0.39)	0.02 Gy (-0.44 - 0.46)	-0.06 Gy * (-1.27 - 0.48)

Lymph nodes	0.01 Gy (-1.09 - 0.91)	0.01 Gy * (-0.11 - 0.36)	-0.09 Gy * (-4.21 - 1.65)	0.08 Gy * (-1.77 - 1.60)

PTV_bladder_	-0.05 Gy * (-3.32 - 0.7)	0.01 Gy (-0.2 - 0.17)	-0.99 Gy * (-8.45 - 0.95)	-0.07 Gy * (-1.21 - 1.34)

The results are displayed as: median (range)

* P-value significant

**Figure 4:**
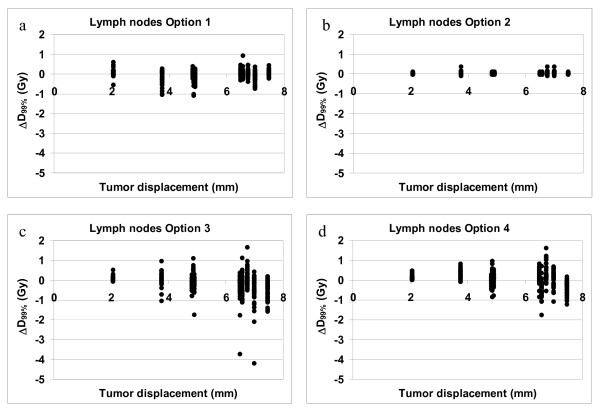
**Lymph node coverage. **These figures show the ΔD_99% _of the lymph nodes versus the tumor displacement vector for the four correction strategies. Note that some of the tumor displacement vector lengths overlap (see table 1)

For the lymph nodes option 1 (no correction) was not statistically significant different from the treatment plan (table 2). When option 2 was applied (correction on bony anatomy), the median ΔD_99% _was 0.01 Gy (range -0.11 to 0.36). This small difference was significant (p < 0.001), because the data were not normally distributed and the positive values were larger than the negative values. Correction based on tumor coverage (option 3) gives the lowest target coverage for the lymph nodes (figure 4).

**Figure 5:**
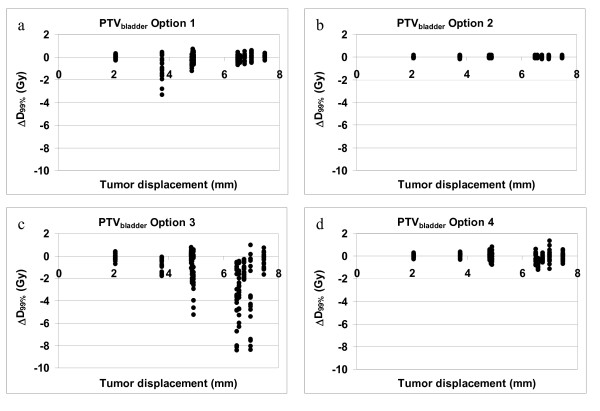
**PTV_bladder _coverage. **These figures show the ΔD_99% _of the PTV_bladder _versus the tumor displacement vector for the four correction strategies. Note that some of the tumor displacement vector lengths overlap (see table 1)

For the bladder as target we analyzed the PTV_bladder_, because the possible volume change is incorporated in the CTV-PTV margin. When option 3 was applied underdosages up to 8.5 Gy can occur (figure 5).

Option 4 is the method that gives the highest coverage in all targets. The difference with the treatment plan never exceeded 2 Gy in all 200 simulations.

### Hot spots

**Figure 6:**
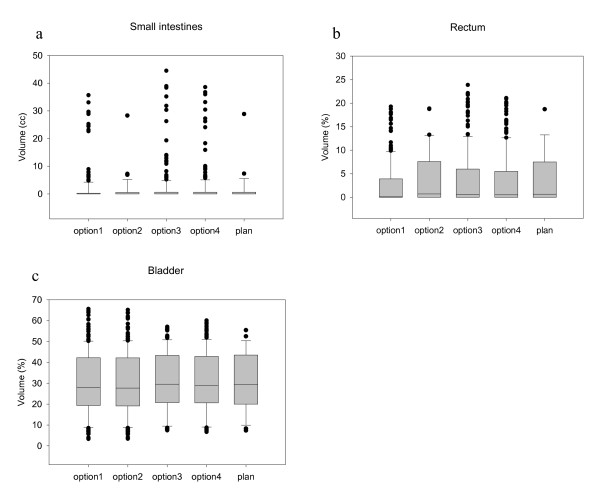
**Hot spots. **Hot spots (volume that receives more than 95% of the prescription dose) of the small intestines, rectum and bladder.

The V_95% _of the small intestines in the treatment plan was very small, the median was 0.0 cc (range 0 - 28.9 cc) and remained small after application of any of the four options (figure 6a). The V_95% _of the rectum in the treatment plan was also small, the median was 0.6% (range 0-18.7) and remained small after application of any of the four options (figure 6b). One patient had undergone rectum resection in the past, so the results for rectum are for 19 patients. The V_95% _of the femoral heads was zero for all options in all patients.

For the bladder as OAR, we determined the hot spots in the same way as for the small intestines and the rectum, except that movement was simulated for the bladder. The V_95% _for the bladder was much larger than that of the other OARs (figure 6c). This was expected because the tumor is a part of the bladder wall. Hence, the PTV overlaps with the bladder. The bladder itself is also a target.

## Discussion

The goal of this study was to investigate the possibilities to separate the treatment plans for the boost and the elective field and move them independently without adverse effects. We found that the dose in all targets (tumor, bladder and lymph nodes) is adequate when position correction was applied separately for tumor and bony anatomy (option 4). This method offers several benefits. First, the table can be corrected with millimeter accuracy. In addition, the margins on both tumor and lymph nodes can be minimized. Moreover, the technique is instantly available for clinical practice.

When the median ΔD_99% _of each treatment option is considered, the difference between all four correction strategies is relatively small (table 2) and the question arises whether position correction is necessary for this patient group. However, it is clear that patients with a large systematic tumor displacement benefit from the application of position correction while position correction for patients with a small systematic tumor displacement does not seem necessary (figures 3 to 5). Unfortunately it cannot be predicted in which patients large systematic tumor displacement will occur. Five out of the ten patients that were used to determine the systematic and random displacement have a tumor displacement vector length of more than 6 mm and those patients will have decreased tumor coverage when no position correction or position correction based on bony anatomy was applied.

The hot spots in the OARs do not significantly change when position correction is applied, indicating that it is a safe procedure.

Hsu et al. found that in case of prostate and lymph node treatment, the dose in the lymph nodes decreased with less than 1% when position correction based on the prostate position was applied [9]. However, they have simulated random displacements only of which the effect will probably cancel out in a treatment of more than 20 fractions. They also show that large dose decreases occur in individual fractions, indicating that the nodal coverage can decrease when large systematic displacements occur. Ludlum et al. and Rossi et al. also conclude that the dose in the lymph nodes decreases if there is a large systematic error in the prostate position [10,11].

Theoretically, the dose in the lymph nodes in option 2 (correction on bony anatomy) and the treatment plan should be exactly the same, because no movement of the lymph nodes was simulated and perfect position correction was applied (figure 4). The minor difference, 0.01 Gy (± 0.03) on average, is caused by the algorithm used for the dose-volume histogram (DVH) calculation. The dose in 10,000 random points in each organ was determined for the DVH of the treatment plan. During the dose calculation of each simulated treatment new random points were generated.

This study only considered translations. Rotations and deformations were neglected. The main goal of this study was to investigate whether the lymph nodes are being irradiated sufficiently when IGRT is applied on the bladder tumor. Translations are the only uncertainties that we can currently correct for in our department. However, we also determined the CTV coverage in this study, without simulating rotations and deformations. Rotations are rather small, as demonstrated by Lotz et al [4]. Present literature on bladder tumor deformation is not unequivocal. Lotz et al found that bladder tumor tissue is very rigid and that only small deformations occur [4]. However, Chai et al found that deformations are small when the tumor is small, but significant deformation was found for tumors with an elongated shape [15]. The possible impact of these deformations on the dose will need to be investigated.

A drawback of daily on-line position verification and correction is an increase in treatment time. During the period required for the image acquisition and evaluation the bladder volume can increase and the tumor might move again. This additional uncertainty should be incorporated in the applied margin, but is expected to be compensated by the increased accuracy. In this study, every simulated tumor displacement and set-up error was corrected for, without applying a threshold. We expect a minimal effect on the dose when displacements of a few millimeters are not corrected, considering the standard applied safety margins. When a robotic couch can be used on a large scale and the radiotherapy technologists do not have to enter the treatment room anymore to correct the table position, carrying out small corrections on a daily basis will become clinically applicable.

## Conclusions

Based on this study we conclude that applying independent position correction on bone for the elective field and on tumor for the boost gives on average the best target coverage, without introducing additional hot spots in the healthy tissue.

## Competing interests

This work was supported by a grant from Elekta.

## Authors' contributions

DR made the IMRT plans for this study, did the simulations and the statistical analysis and is the main author of the manuscript. JK gave support with treatment planning and the design of the study. RP and AB provided the software for the simulation. MH delineated the structures necessary for treatment planning. AB gave support with the statistics. AB, CK and JK were the senior researchers and provided coordination during the study. JK, RP, MH, CK and AB reviewed the manuscript. All authors have read and approved the manuscript.
